# Independent investigator incubator (I^3^): a comprehensive mentorship program to jumpstart productive research careers for junior faculty

**DOI:** 10.1186/s12909-018-1290-3

**Published:** 2018-08-06

**Authors:** John Paul Spence, Jennifer L. Buddenbaum, Paula J. Bice, Julie L. Welch, Aaron E. Carroll

**Affiliations:** 1 Department of Pediatrics, Section of Pediatric and Adolescent Comparative Effectiveness Research, Indiana University School of Medicine, Health Information and Translational Sciences (HITS) Building, Suite 2030, 410 West 10th St., Indianapolis, IN 46202 USA; 2 Department of Emergency Medicine, Indiana University School of Medicine, Indianapolis, IN 46202 USA

**Keywords:** Mentoring, Junior faculty, Professional development, Translational research

## Abstract

**Background:**

In the highly competitive environment of academic medicine, junior faculty investigators face high attrition rates due to challenges in finding effective mentorship, securing grant funding, and obtaining resources to support their career development and research productivity. The purpose of this study was to describe the centralized, cost-sharing design of the Independent Investigator Incubator (I^3^) program as a novel approach to junior faculty mentoring and to evaluate quantitative outcomes for program improvement.

**Methods:**

In September 2014, the I^3^ pilot program, a comprehensive mentorship program targeting junior faculty pursuing research careers, was launched. Participants included junior faculty during the crucial first three years of their research careers or during their transition from career development awards to more independent research. Following initial screening, the I^3^ mentees were paired with a senior faculty “super-mentor” with expertise in either basic science or clinical research. Mentees were provided with robust traditional one-on-one mentoring, targeted feedback from a super-mentor review committee, as well as biostatistician and grant writing support. To assess the effectiveness of the I^3^ program, we tracked outcome measures via baseline and 12-month mentee surveys. Data collected assessed program diversity, mentee self-assessments, evaluation of the mentoring relationship, scholarship and productivity metrics. Raw data were analyzed using a paired t-test in Excel (*P* < 0.05).

**Results:**

Results of the baseline mentee self-assessment survey found that the I^3^ mentees indicated common “perceive deficits” including navigating the organizational and institutional culture, clear direction in achieving promotion and tenure, among others. When baseline mentee survey responses were compared to 12-month responses, we identified strong “perceived growth” in categories, such as Research and Interpersonal Skills and Career Development Skills. Further, productivity metrics at 12-months revealed that roughly 80% of I^3^ mentees successfully published a manuscript(s). The I^3^ program has helped generate roughly $12.1 million dollars in investigator-initiated funding after two years in the program.

**Conclusion:**

The I^3^ program allows for shared costs between institutions and increased availability of successful subject matter experts. Study results imply that the I^3^ mentoring program provides transformative mentorship for junior faculty. Using our findings, we developed courses and an annual “snapshot” of mentee performance for mentors.

**Electronic supplementary material:**

The online version of this article (10.1186/s12909-018-1290-3) contains supplementary material, which is available to authorized users.

## Background

Mentoring in academic medicine provides an important mechanism for faculty to develop the necessary skillsets to succeed in a highly competitive environment by improving faculty career satisfaction, retention, research productivity, and professional success [1–6]. While the mentoring relationship is a foundation of graduate and medical education, as well as postdoctoral training, the application of mentoring to junior faculty in academic medicine is often more ambiguous. The transition from a trainee to a junior faculty appointment for both basic-scientists and clinician-scientists marks a point at which the acquisition of independence begins to supersede the importance of conducting elementary science, developing technical capabilities, or acquiring clinical knowledge [7].

Effective mentorship is an essential element to supplement a research faculty’s personal and professional development by providing guidance through the mentor’s expertise, sponsorship, and institutional knowledge. Thereby, the mentee develops professional competency in career planning, communication skills, research and scholarship skills, managerial and leadership skills, negotiating and networking skills, and navigating the institutional culture [2, 8, 9]. As many of these skills are not explicitly taught, junior faculty rely on a mentor to help attain these additional skillsets.

For research faculty, success is often defined by the ability to transition from trainee to early career awardee to independent investigator [10]. The urgency of this transition is particularly important in the high-stakes environment of academic medicine where appointees often enter into short-term contractual agreements or face competing demands of clinical and teaching workloads. The role of mentorship to facilitate the research career path of a junior faculty member is most crucial during the early period (i.e., the first three to five years), when otherwise talented investigators are lost to competing employment opportunities, in part, due to their inability to successfully navigate the academic and funding environment [11–13]. This “academic attrition” threatens to undermine the overall talent-base of academia itself [14].

As National Institutes of Health (NIH) budgets have been markedly reduced over the past decades, faculty have faced decreasing success rates in securing independent funding. For instance, the success rate for obtaining an R01-equivalent grant has fallen steadily from 33% in 1976, to 27% in 1996, to 20% in 2016 (NIH Research Portfolio, https://report.nih.gov/index.aspx). In a 2015 article, Yin and colleagues reasoned that the decreasing pipeline of clinician-scientists in clinical and translational research is due in large part to the mounting challenges in transitioning from early career (K-type) to independent (R-type) funding, citing mentoring as “the single most important factor” in transition from K to R [15–17]. However, other factors likely contribute to academic attrition, specifically limited startup funding and FTE for research. Therefore, institutions who wish to compete for top talent must recognize the urgency of supporting the transition to independence, particularly in the current high-stakes research environment of today.

Potential barriers that impede the implementation of effective mentoring in many departments or divisions in academic institutions include the lack of an experienced mentor pool [15–17] and inadequate resources and time to support the development of their research faculty [18]. This scenario is especially true for clinical departments trying to spur lines of research. Additionally, some departments and institutions face barriers such as the geographic disbursement of faculty, invisible silos between units, and the heterogeneity of disciplines within the institution. Further, unique issues exist for medical centers that wish to foster and fund clinician-scientists in clinical and translational research. Though highly adept at concepts of critical needs facing patient care, these junior scientists often lack formal training in conducting research and rely on mentorship and support services that are not often available [10].

One suggested solution is to seek research mentorship from other disciplines in order to expand the mentor pool of senior investigators [16]. Rather than relying on each department to provide mentorship from within, a more efficient strategy might be for institutions to centralize resources and gather excellent mentors from across the institution, thereby concentrating mentorship among those faculty who are particularly suited to the task. This interdisciplinary mentorship model is feasible, as the fundamental principles of research mentoring are universal, and has been successfully employed at other academic medical institutions to mentor junior faculty investigators [19–21]. The literature offers two examples of successful mentoring programs that targeted junior research faculty (i.e., University of Utah and Duke University) [20, 21]. These mentoring models employed similar elements to our I^3^ Mentoring Program, which included targeting early career research faculty and providing multidisciplinary mentorship, focused grant writing development, and access to centralized support services within the institution [20, 21]. Both programs also demonstrated increased NIH funding rates for their junior faculty mentees.

The purpose of this study was to describe the cost-sharing, centralized design of the I^3^ program as a novel approach for junior faculty at IUSM. In addition, this study was conducted to “evaluate” quantitative outcomes specially focused on mentee input that could be utilized to improve the (I^3^) program both at administrative level and to provide more targeted mentoring. We report the 24-month pilot outcomes of the Independent Investigator Incubator (I^3^) Program focused on short-term and medium goals. Measures of effectiveness included mentee self-assessments, acquisition of professional skills, evaluation of the mentoring relationship, and productivity metrics (e.g., grant submissions, acquired funding, and publications). This research identified quantitative variables (e.g., perceived deficits) provided administrative insight for program improvement and was employed to develop a novel tool for more “targeted mentoring”.

## Methods

### I^3^ program structure

**Fig. 1:**
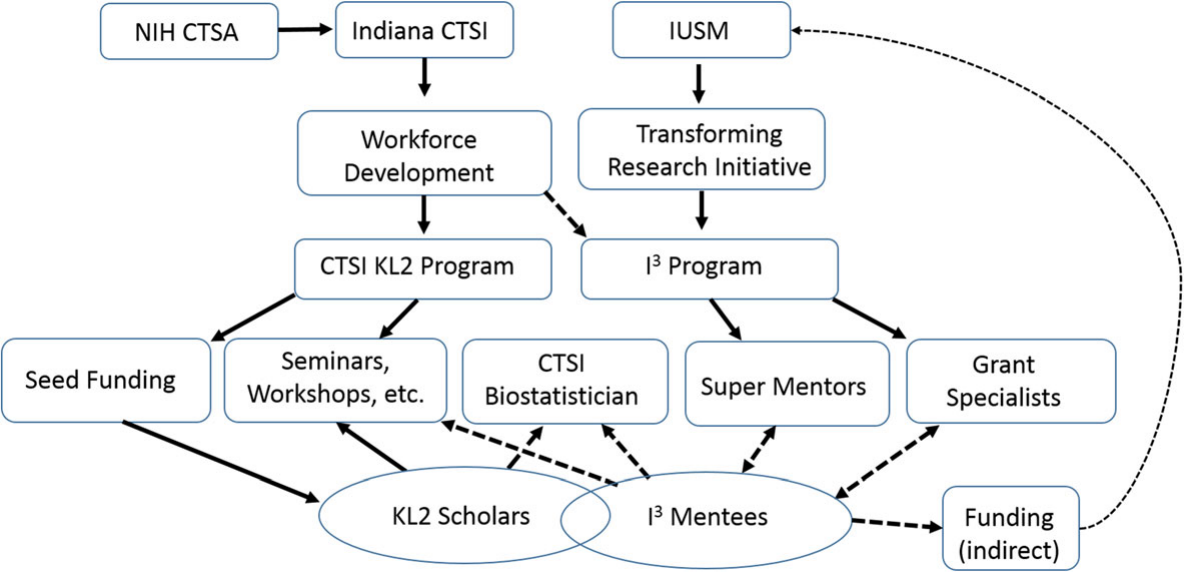
Centralized, cost-sharing model of I^3^ mentoring program. The I^3^ program tapped into existing resources developed for the existing CTSI KL2 program, a CTSA-affiliated program. The interinstitutional cost-sharing model decreases the financial burden associated with the I^3^ program

In September 2014, the Indiana University School of Medicine (IUSM) Transforming Research Initiative (TRI) in collaboration with the Indiana Clinical and Translational Sciences Institute (CTSI) established the Independent Investigator Incubator (I^3^) Program. Indiana CTSI is a “hub” of the Clinical and Translational Science Awards (CTSA) Program. CTSA supports a national network of medical research institutions that work together to improve the translational research process to get more treatments to more patients more quickly. The hubs collaborate locally and regionally to catalyze innovation in training, research tools and processes. The primary goal of the I^3^ Mentoring Program is to facilitate the transition of high potential junior faculty researchers into independent investigators by removing as many barriers as possible and ultimately increasing their grant funding success. The I^3^ Mentoring Program employs a cost-sharing, centralized design that concentrated one-on-one mentorship from a senior faculty “super mentor,” feedback from a “super mentor” committee, mentoring resources, career development workshops, a professional grant writer and biostatistical support (Fig. 1). The I^3^ Mentoring Program is designed to synergize with any existing mentoring relationship at the departmental level. Each I^3^ mentee-mentor pair agrees to make a dedicated commitment to the mentee’s personal and professional success. In addition, a grant writing team works closely with the I^3^ mentees to support the grant application process, from drafting and editing sections of proposals, to assembling grant packages, and submitting materials. This team also assists in the development of new concept proposals and facilitates the successful publication of manuscripts.

### Participants

“Super mentors” were identified and recruited from across the institution by the Associate Dean of Research Affairs based on their successful track records of mentorship, sustained funding, and productive research careers. These “super mentors” agreed to serve on an oversight committee to screen mentee applicants, mentor individual mentees in the program, and meet on a regular basis to review the progress of the program. Unlike many mentorship programs, I^3^ mentors are compensated for their efforts with 5% full-time equivalency (FTE) for each mentee, up to 15% FTE. “Super mentors” were provided with professional development opportunities including online CTSI mentoring training modules [22], the institution’s annual faculty mentoring symposium, resources to support effective mentorship via the IUSM Faculty Mentoring Portal [23], and expectations to support their mentoring relationships. The expectations included, but were not limited to: (1) be available to meet regularly with the mentee, (2) set up a mentorship panel, (3) review and provide feedback on goal setting based on the mentee’s self-assessment and Individual Development Plan (IDP), (4) provide oversight and guidance on the mentee’s research, writing, and grant submission activities, (5) utilize the tools in the Mentor Toolkit as necessary (e.g., mentoring agreements, meeting checklists, IDP), (6) plug the mentee into mentoring resources, career development opportunities, and research services (i.e., peer mentoring, biostatistician, grants administration).

I^3^ Mentees were recruited from across the institution via institutional newsletters, email alerts, and online departmental communications. Mentee applications were accepted on a rolling enrollment basis. Junior faculty applicants were screened and selected by the “super mentor” committee based on their academic achievements, area of research, and potential to become an independent investigator. The screening criteria targeted junior faculty during the crucial first three years of their research careers (i.e., at the stage to apply for an early career development award) or during their transition from career development award to investigator initialed funding. The I^3^ program required that the applicants had a departmental commitment to protected time for research, typically 50–75% FTE. This requirement is important to address the concern that clinical faculty require adequate protected time from their respective department to conduct research. I^3^ mentees were then matched to a specific “super mentor” with expertise in either basic science or clinical research.

Mentees were provided with comprehensive support including: (1) robust one-on-one mentorship and targeted feedback from the “super mentor” review committee. (2) assistance from “super mentor” to set up a mentorship panel, (3) career development workshops through the CTSI and IUSM on a variety of topics (e.g., creating and implementing your IDP, mentoring skills, research/budgeting skills, scientific and grant writing skills, work-life balance, conflict management, and more), (4) mentoring resources and training including the Mentee Toolkit from the IUSM Faculty Mentoring Portal. The I^3^ pairs also were provided with structured expectations (e.g., meet on a regular basis, complete and review mentee self-assessment and IDP, and participate in program surveys). The present study was accepted as Exempt (1) Category 1: Educational Research Conducted in Educational Settings; therefore, informed consent has been waived by the Indiana University Institutional Review Board (IRB) for this research (Study #: 1601639726).

### Methods of administrative review

**Fig. 2:**
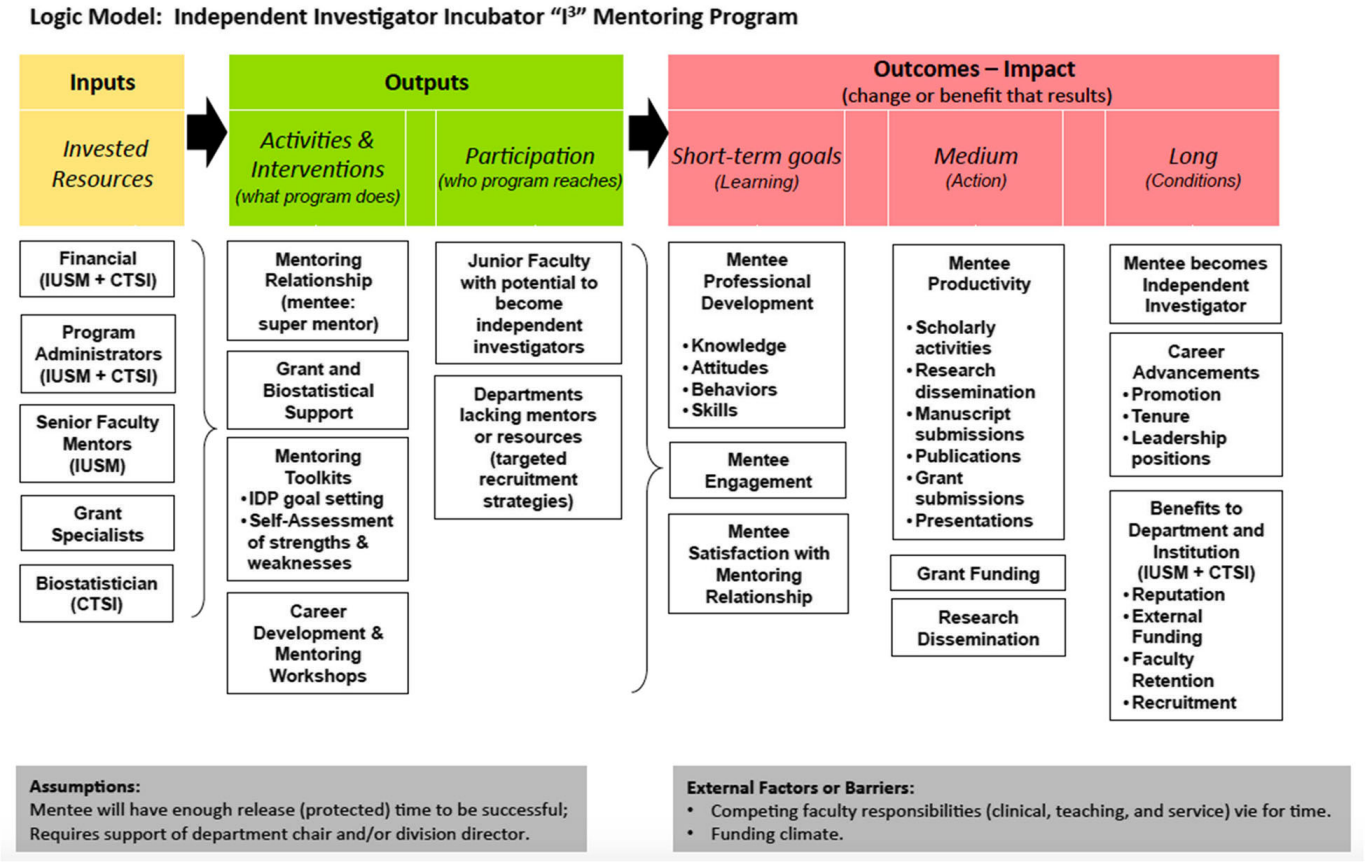
Logic model for the I^3^ mentoring program. The figure demonstrates the logical framework that was utilized to develop and to evaluate the effectiveness of the I^3^ mentoring program

This study provides an initial “administrative review” of mentee performance focusing on short-term goals and outcomes at 12 months (short-term) and funding at 24 months (medium). The logic model (Fig. 2) provides the proposed the inputs, outputs and outcomes related to the I^3^ program. Mentees were required to complete a Mentee Self-Assessment Survey and an electronic I^3^ Mentoring Program Survey. The Mentee Self-Assessment Survey was designed and adapted via expert opinion from University of California, San Francisco Faculty Mentoring Program. This 40 item self-evaluation tool was initially implemented at IUSM to assist the junior faculty mentee in assessing their professional strengths and weaknesses (Additional file 1: Figure S1). This survey measures their self-reported knowledge, skills, or abilities in seven categories of skillsets reflecting important indicators of faculty professional competence: (i) Mentoring Relationship Skills, (ii) Research Skills, (iii) Scholarship Skills, (iv) Leadership and Management Skills, (v) Interpersonal Skills, (vi) Career Development Skills, and (vii) Teaching Skills. Each item is scored on a Likert scale of 1 to 5, where 1 indicated “needs improvement” and 5 indicated “highly proficient”. It was an expectation that every I^3^ mentee complete this self-assessment in conjunction with their IDP and then review these documents with their mentor in order to gain feedback in developing and strengthening their skills. The mentors were provided with both the baseline and 12- month self-assessment results in order to gage mentee progress and inform future development needs. In this study, we compared Mentee Self-Assessment Survey data at baseline (time zero) and 12 months as measures of “Perceived Deficits” and “Perceived Growth” as an indicator of short-term outcomes (Fig. 2). “Perceived Deficits” were defined as scores less than 3 (scale of 1 to 5) at baseline. “Perceived Growth” was defined as the positive change in scores for specific skills from baseline to 12 months.

The I^3^ Mentoring Program Survey was a 44-item electronic assessment tool designed through expert opinion using an iterative process to reach consensus via a Delphi method. The I^3^ Mentoring Program Survey collected outcome measures regarding mentee professional growth and development, productivity, as well as the overall effectiveness and impact of the I^3^ Mentoring Program. For this purpose, data collected from the Mentoring Program Survey included questions concerning the mentee’s professional development (i.e., knowledge, attitudes, behaviors and skills), satisfaction with the I^3^ Mentoring Program and the mentoring relationship, academic productivity measures (e.g., grant submissions, acquired funding, presentations, and publications), and career status and advancement (e.g., degrees, rank, track, titles, leadership positions). In addition, we collected data on grant funding awarded at 12 and 24 months to better address funded grants that had been largely conceived or written prior to the start of the program.

### Data collection and analysis

Data were collected and managed using Research Electronic Data Capture (REDCap) tools hosted at Indiana University, which is a secure, web-based application for electronic surveys and databases [24]. Data analysis was performed in Excel with differences expressed as percentages in the Results. For “Perceived Growth”, raw data (Likert scale) were analyzed using a paired t-test in Excel (*P* < 0.05).

## Results

### I^3^ mentoring program was composed of a diverse group of junior faculty

**Fig. 3:**
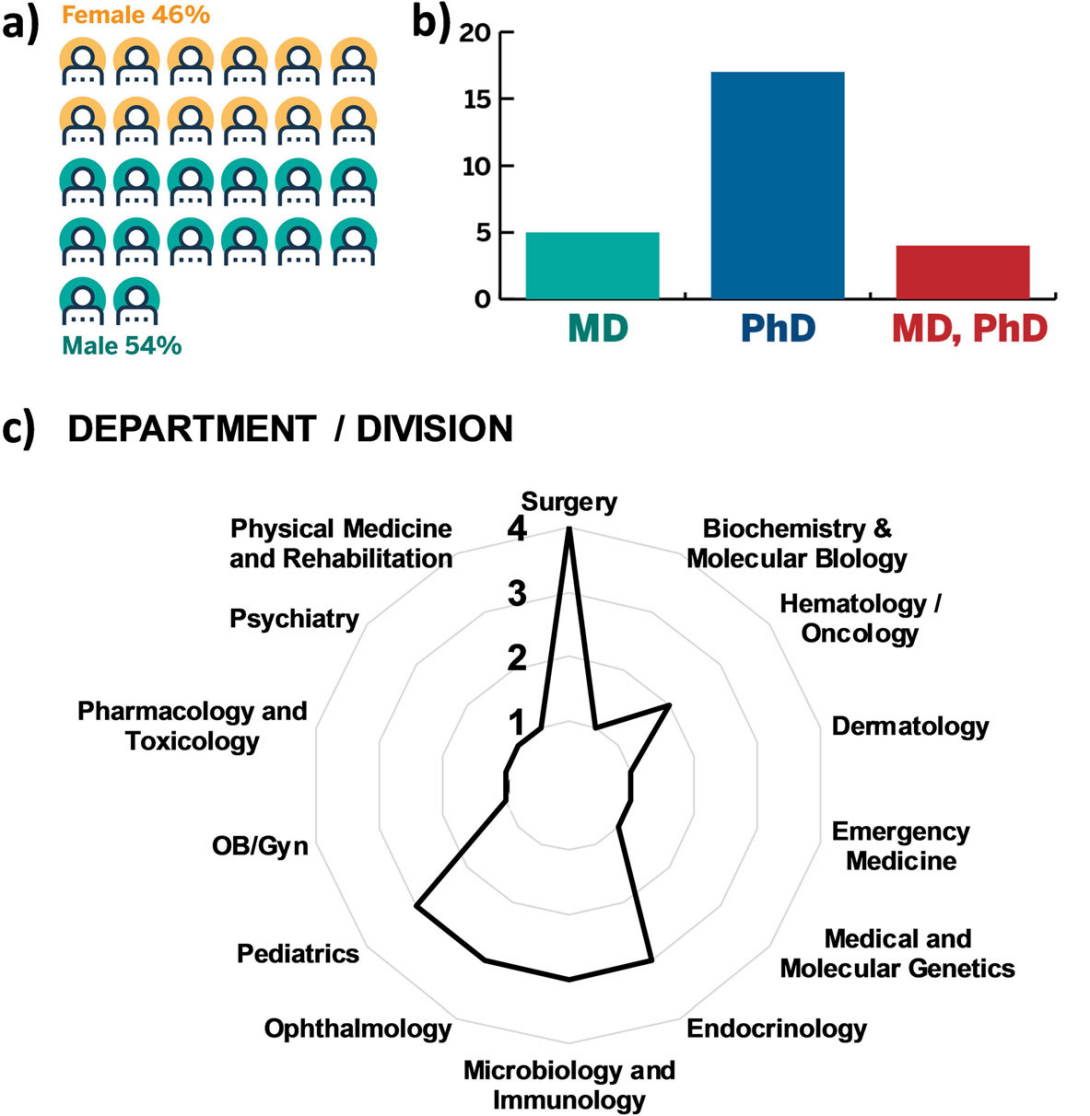
Demographic and academic diversity of the I^3^ mentoring program. Upon entering the I^3^ mentoring program, the mentees were asked to complete a baseline survey. The collected survey data were directly entered by the participant into a secure, online REDCap database. The figures depict demographics and career-related information including sex (**a**), terminal degree (**b**), and department / division (**c**). The graphs indicate percentages of I^3^ mentees at 12 months (*N* = 26)

The 12- month pilot study included 26 mentees who were mentored by 10 “super mentors”. All 26 mentees completed the Mentee Self-Assessment and I^3^ Mentoring Program Surveys collected at baseline (time zero) and 12 months. The I^3^ mentees represented a diverse group of research faculty based on sex, ethnicity, terminal degree, academic track, and discipline. For instance, the I^3^ Mentoring Program recruited a similar number of males (54%) and females (46%) (Fig. 3a) and consisted of both PhD-scientists and clinician-scientists (Fig. 3b). Notably, fourteen departments were represented in the I^3^ Mentoring Program (Fig. 3c).

### Mentee self-assessment survey identified specific “perceived deficits”

**Fig. 4:**
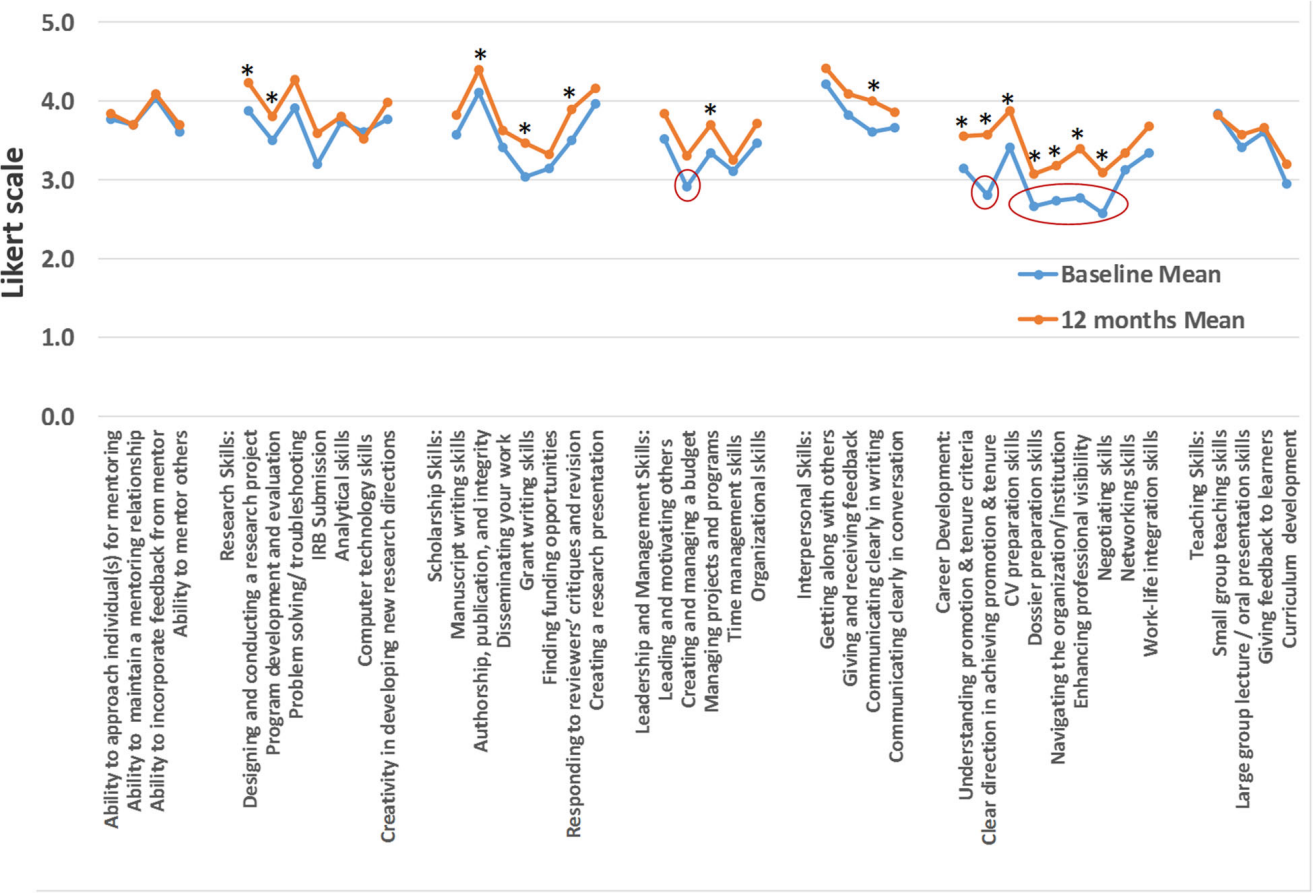
Mentee self-assessment survey shows “Perceived Deficits” and “Perceived Growth” after 12 months. The graph depicts averages from the raw data obtained from the mentee self-assessment survey (Likert scale) (N = 26). “Perceived Deficits” were defined as responses for specific skills that were less than 3 (scale of 1 to 5), where 1 indicated “needs improvement” and 5 indicated “highly proficient”. “Perceived Growth” was defined as a positive change in scores (scale of 1 to 5) from baseline to 12 months for specific skills. Raw data were analyzed using a paired t-test (*P* < 0.05)

On the baseline (time zero) Mentee Self-assessment Survey, mentees identified six professional skills as “Perceived Deficits”. Most notably, over 40% of I^3^ mentee indicated deficits in “negotiating skills,” “dossier preparation skills” and “navigating the institutional culture.” In addition, over 30% indicated deficits in “creating and managing a budget”, “clear direction in achieving tenure,” and “enhancing professional visibility,” (Fig. 4). Comparing these baseline results to the 12-month results, the I^3^ mentees indicated improvements in all “perceived deficits” items. However, “dossier preparation skills” and “creating and managing a budget” were still identified as skillsets where many I^3^ mentees noted a deficit at 12 months. Together, the results demonstrated potential weaknesses shared by I^3^ mentees, specifically related to “Career Development Skills” and “Leadership and Management Skills.” These deficits can be administratively targeted to improve the mentoring program.

### Mentee self-assessment survey identified key categories associated with “perceived growth”

Three categories were identified as areas of “perceived growth” by mentees during the first year of the I^3^ Mentoring Program, including “Research Skills,” “Communication Skills” and “Career Development Skills” (Fig. 4). Notably, specific skill sets related to “designing and conducting research,” “program development and evaluation”, “authorship, publication, and integrity”, “grant writing skills,” “responding to reviewers’ critiques”, “managing projects and programs” and “communicating clearly in writing” were identified as areas of research-related growth, while “understanding promotion and tenure criteria”, “clear direction in achieving promotion and tenure,” “CV preparation skills”, “navigating the organizational culture”, “enhancing professional visibility” and “negotiating skills” were indicated as key career development growth. These findings provide initial evidence that the I^3^ Mentoring Program is effective at addressing the short-term goal (learning).

### I^3^ mentoring program survey showed positive indicators of success

**Fig. 5:**
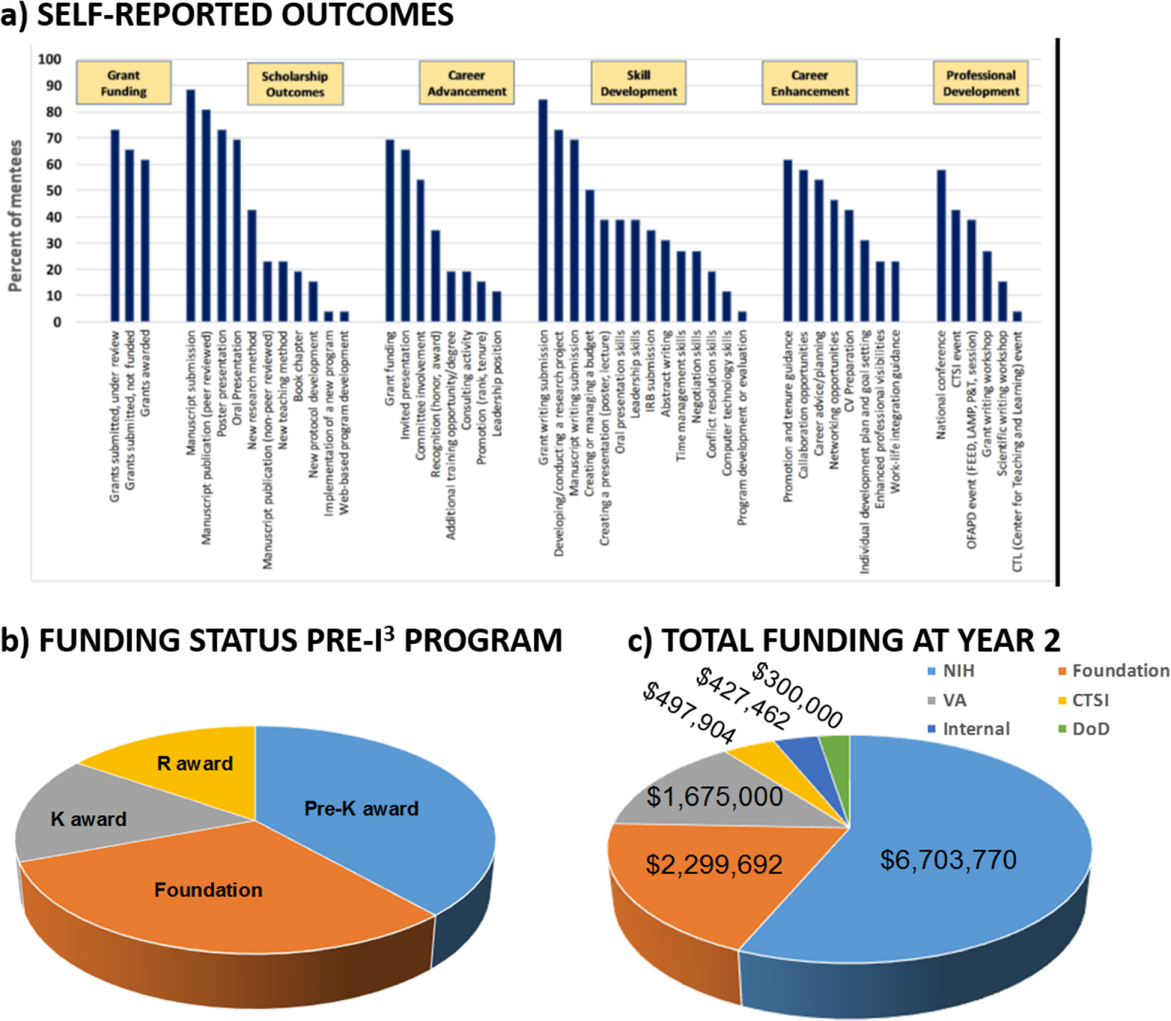
Outcomes assessment identifies key indicators of success for I^3^ mentees. The Outcomes Assessment was utilized to assess career-related growth and their academic achievements (e.g. publications / funding). **a** Outcomes data from Outcomes Assessment at 12 months – based on the percentage of total I^3^ mentees, **b** Funding status prior to entering the I^3^ program, **c** Total acquisition of investigated-initiated funding (dollars) at 24 months (N = 26). NIH - National Institutes of Health; VA - The United States Department of Veterans Affairs; Internal - Indiana University School of Medicine; CTSI - Indiana Clinical and Translational Sciences Institute

In this study, we compared Mentee Self-Assessment Survey data at baseline (time zero) and 12 months as an indicator for short-term outcomes / learning (Fig. 2). On the 12-month I^3^ Mentoring Program Survey, I^3^ mentees expressed that they benefited from multiple career development tools and mentoring advice. In addition, all mentees were satisfied with their mentor pairing based on the mentor’s “availability” and “valuable feedback,” and all mentees wanted the mentoring relationship to continue. During the first year, all of the mentees were actively engaged with the majority of mentees meeting with their mentor either weekly or biweekly. In terms of productivity outcome measures, the majority of I^3^ mentees reported scholarship outcomes and dissemination of their research via manuscript submission (88%), manuscript publication (80%), poster presentation (73%), and oral presentation (69%) (Fig. 5a). The I^3^ mentees reported a total of 81 publications at 12 months, an average of 3.1 publications per mentee. In addition, I^3^ mentees reported high rates of “grant writing submission” (85%) and “grant funding” (69%). The initial funding status of I^3^ mentees is shown in Fig. 5b. Funding acquisition was not associated with pre-I^3^ funding status. I^3^ mentees were awarded roughly $6.9 million dollars after one year in the program and $12.1 million after 2 years (Fig. 5c). Notably, the median funding level changed from $40,000 to $268,000 comparing year one to total funding acquisition at year 2, respectively.

## Discussion

In this manuscript, we report the pilot outcome data for the program that was utilized for initial program evaluation and improvement. During its first year, the I^3^ mentoring program recruited a diverse group of junior faculty based on sex, ethnicity, academic degree, and discipline. Notably, using mentee self-assessments (at baseline and 12 months), we identified important mentee perceptions of their skills for a variety of important domains where I^3^ mentees indicated “perceived deficits,” all of which improved after one year. In addition, we found that the I^3^ program positively impacted the mentees’ self-perceived professional growth and competence in domains, such as research and career development. Moreover, we found that the mentees are satisfied with their mentor pairing and desire the mentoring relationship to continue. Finally, we report productivity outcomes, such as publications and grant funding.

The I^3^ program utilizes a unique centralized mentorship assignment process for “super mentors” that allows for shared costs and increased availability of successful subject matter experts. The I^3^ program was designed to provide intensive mentoring and other support that synergizes with and promoted pre-existing departmental mentoring in a large medical research environment, such as IUSM. Unlike the centralized mentoring programs that targeted junior research faculty at the University of Utah and Duke University [20, 21], the I^3^ program was designed as a three-year program with the possibility of extension based on mentee engagement, productivity, and career development needs. This is important because the ultimate goal of the I^3^ program is to espouse “independent investigators” with R01-type funding, and junior faculty with pre-K or K-type funding may require additional support. In addition, the cost-sharing model of I^3^ program is unique in that administrative and mentoring resources are shared between Indiana CTSI and IUSM (Fig. 1). The interinstitutional cost-sharing model decreases the financial burden associated with the I^3^ program. Importantly, the I^3^ program tapped into existing resources developed by the CTSI KL2 program, a CTSA-affiliated program that provides seed funding and career development resources to pre-K faculty.

Since 2008, the “curriculum” for the CTSI KL2 scholars has consisted of 1–2 talks/workshops each semester designed to cycle over 2 years and then repeat. The topics have included: (i) Mentor Panel (former KL2s giving advice), (ii) Work-Life Balance, (iii) Conflict Management, (iv) Scientific Writing (v) Keeping Tabs on your grant budget, (vi) Factors predicting success and failure for K to R transitions, (vii) Team Science, and (viii) Individual Development Plans. The program also provides writing workshops. Thereby, the I^3^ program increases the impact of the CTSI-related workforce development component by including junior faculty who might not fit into the classic pre-K to K-type faculty transition. Importantly, the CTSI KL2 program is more selective reaching 56 junior faculty from 2008 to 2018, while the I^3^ program has mentored 61 junior faculty from the fall of 2014 to 2018.

The I^3^ program recruited a core group of accomplished research mentors and compensated them with financial salary support. Freel et al. employed a similar method. This element attempts to mitigate a potential cost-benefit issue facing the mentoring relationship by incorporating a means by which to fund our mentors, thus tangibly recognizing and rewarding their time and expertise. For example, unlike graduate and postdoctoral trainees where the mentor-mentee relationship is often mutually beneficial, the mentoring relationship following the transition from trainee to junior faculty changes based on the cost-benefit ratio [25]. There are fewer tangible incentives for mentoring junior faculty from the mentors’ perspective. A recent national survey of academic internal medicine mentors found that less than half of mentors (48%) received funding for mentorship. Interestingly, funded mentors were more likely than unfunded mentors to take on mentees (mean of 8.3 vs 5.1, respectively) [26]. This indicates that while the literature is full of articles acknowledging the importance of mentorship in academic medicine, the majority of mentors are not compensated for mentoring.

Next, we asked the following program evaluation question. “Can mentee outcomes and self-assessment be utilized to identify quantitative outcomes to evaluate the program at the administrative level and to provide more targeted mentoring?” The logic model (Fig. 2) provides the inputs, outputs and outcomes related to the I^3^ program. As the program is intended to be a three year process for junior faculty development, this study examined a variety of important domains (short-term goals - learning), as well as products such as papers, grants and other measures of productivity (medium goals). The 12-month mentee survey consisted of (i) mentor evaluation, (ii) mentee outcomes evaluation and (iii) self-assessment survey. Outcomes and self-assessment were used to gain insight regarding whether this intervention was effective in spurring a variety of important domains, as well as products such as papers and grants. In addition, this study was conducted to “evaluate” quantitative outcomes specially focused on mentee input that could be utilized to improve the I^3^ program both at administrative level and to provide more evidence-based, targeted mentoring.

Using mentee self-assessment surveys, we identified six “perceived deficits” in skillsets related to “career development” and “leadership and management” skills, all of which improved after 12 months in the program. However, many still noted deficits in procedural skillsets, such as “dossier preparation” and “creating and managing a budget.” This information provides administrative insight for the development of novel interventions, specifically targeting introspective input from the mentees themselves. Identifying these global deficits has enabled the I^3^ mentoring program to strategically target these deficits with additional seminars, workshops and courses. Indeed, a grant writing course is now being developed for junior faculty at IUSM. Similar to grant writing, the mentee can be explicitly taught how to create and manage a budget.

In addition to identifying deficits, we found that the I^3^ mentoring program fostered growth and development in key areas essential for building professional competence for research faculty. When evaluating “perceived growth” at 12 months, the greatest percentage of individuals indicated improvements in areas related to research and communication skills as well as career development skills. Some examples included grant writing, IRB submission, budget preparation, and curriculum vitae (CV) preparation. Others included communicating in conversation and writing, understanding authorship and publication, as well as designing and conducting research.

While the academic procedural skillsets are necessary, the progression from junior investigator to independent investigator is dependent on the mentee’s ability to navigate the academic environment and integrate into the fabric of the academic institution. For instance, the ability to negotiate is critical for collaborative work with other scientists. Further, understanding promotion and tenure is more than developing a timeline and checklist of independent achievements. It requires a longitudinal plan that encompasses individual goals in context with a broad understanding of the needs and opportunities at the departmental and institutional levels along with keen insight into the people and processes that identify those needs. Self-assessment findings were used to gain administrative insight regarding whether this mentoring intervention was effective in spurring a variety of important domains for administrative program improvement specifically short-term goals related to learning (Fig. 2).

Next, we examined outcomes and products such as papers, grants and other measures of productivity (medium goals). We found that the I^3^ program impacted other key outcome measures including scholarship, research dissemination and acquisition of funding. Indeed, roughly 80% of the mentees indicated that they successfully published a manuscript(s) during their first year in the program, and roughly the same percentage of individuals presented their work (i.e., oral or poster). Notably, I^3^ mentees were awarded roughly $6.9 million dollars after one year in the program and $11.85 million after 2 years with a median funding level change from $40,000 to $268,000, respectively. While many of the faculty have a substantial trajectory coming into the program (Fig. 5b), all of the funded grants submitted during their time in the program, and nearly all proposals were developed with the help from grant writing support, thereby providing a positive indicator of program success. The diversified distribution of funding sources provides insight into mechanisms that may be appropriate for junior investigators to pursue.

Based on publications and funded grants (medium goals), four mentees were identified who did not seem to benefit from the program. Interestingly, all of these individuals experienced life changing events that were likely associated with their gaps in research productivity, including impending relocation and family leave. Since the inception of the I3 program, another factor that has also emerged as a potential obstacle for success includes limited program engagement due to distance (i.e., satellite campuses). We are currently working on strategies to improve engagement for individuals from satellite campuses by increasing mentor- and program-initiated contact and promoting the establishment of recurring meetings between the mentee and mentor. Because the I^3^ program has limited resources, it is important to identify and address any obstacles that might hinder the mentoring process and overall success of the program.

Another goal of this research was to develop a supplemental tool for more targeted mentoring. The concept was to develop a short report (1 page) that provides the mentors with a “snapshot” of assessment of mentee performance, mentee indicators of success (publications, funding), and mentee changes in career-related perception over time. This snapshot is intended to supplement mentor impressions of the mentee and to identify perceived deficits that can be addressed. An example of this report is provided in the Additional files (Additional file: 2 Figure S2).

We acknowledge several important limitations to the present study. These limitations include a single site, pilot data, and a relatively low number of participants. This study is not well-suited for comparative research related to the relative contribution or impact of specific metrics between institutions. While this descriptive study is not well-controlled, the study still provides tangible outcomes for administrators that develop mentoring programs in similar academic settings, especially productivity metrics. However, it should be evident if junior faculty at other institutions show similar levels of productivity. If they are underperforming, the institution might consider developing a centralized mentorship program similar to the I^3^ program.

While more complex multifactorial analyses might identify specific components/activities that contribute to outcomes, this study focused on self-report and outcomes as a potential indicator of the effectiveness of the I^3^ program itself. The analyses were limited by the sample size (*n* = 26). In addition, it is impossible to isolate the effects of the program from other resources that are provided by IUSM. IUSM provides many resources to spur junior faculty development at this institution. These include, but are not limited to, internal funding mechanisms, seminars, workshops, etc. It is very possible that these resources also contributed to the success of junior faculty. Further, the program elements were not systematically evaluated to determine their relative value. Therefore, mentee input regarding the impact of specific elements of the program would be interesting to include for future program evaluation.

## Conclusions

The I^3^ Mentoring Program demonstrated indicators of success based on the mentee’s satisfaction with their mentoring relationship, perceived professional growth, and productivity outcome measures, such as manuscript publication and funding acquisition. In addition, this study provided important information for program improvement focusing on more global interventions (e.g., course development) as well as the creation of a novel mentor-specific tool (i.e., mentee snapshot). Ultimately, the cost-sharing, centralized design of the I^3^ mentoring program might serve as an effective, cross disciplinary mentorship for other academic institutions to utilize in order to jumpstart productive research careers for their junior faculty.

## Additional files

Additional file 1: **Figure S1.** The Mentee Self-Assessment Survey. The Mentee Self-Assessment Survey was designed and adapted via expert opinion from University of California, San Francisco Faculty Mentoring Program. This 40 item self-evaluation tool was implemented at IUSM to assist the junior faculty mentee in assessing their professional strengths and weaknesses. (PDF 2981 kb)

Additional file 2: **Figure S2.** Snapshot of mentee performance for I^3^ mentors. A short report (1 page) was developed to provide the mentors with a “snapshot” of assessment of mentee performance over time in order to supplement mentor impressions of the mentee and to identify perceived deficits that can be addressed. (PDF 6781 kb)

## Abbreviations

CTSA: Clinical and Translational Science Awards
CTSI: Indiana Clinical and Translational Sciences Institute
CV: Curriculum Vitae
FTE: Full-time Equivalency
I^3^: Independent Investigator Incubator
IDP: Individual Development Plan
IRB: Institutional Review Board
IUSM: Indiana University School of Medicine
NIH: National Institutes of Health
REDCap: Research Electronic Data Capture
TRI: Transforming Research Initiative
VA: The United States Department of Veterans Affairs
