# Supplementary material for: Independent investigator incubator (I3): a comprehensive mentorship program to jumpstart productive research careers for junior faculty
Source: BMC Med Educ. 2018 Aug 6;18:186. doi: 10.1186/s12909-018-1290-3 (PMC6080403; doi:10.1186/s12909-018-1290-3)
Supplement: Supplementary file 1 — Figure S1. The Mentee Self-Assessment Survey. The Mentee Self-Assessment Survey was designed and adapted via expert opinion from University of California, San Francisco Faculty Mentoring Program. This 40 item self-evaluation tool was implemented at IUSM to assist the junior faculty mentee in assessing their professional strengths and weaknesses. (PDF 2981 kb) [file 12909_2018_1290_MOESM1_ESM.pdf]

Mentee Self-Assessment Worksheet

This self-evaluation is designed to assess your skills, abilities, strengths and weaknesses in your development as a academic faculty member. Complete this in conjunction with your IDP (Individual Development Plan) and share with your mentor. Request feedback and guidance in developing and strengthening your skills. It may be helpful to do a follow up assessment in order to evaluate progress.

Rate on a scale of 1 to 5:
1 = Needs improvement
5 = Highly proficient

Mentoring Relationship Skills:

As a mentee: ability to identify and approach individual(s) for mentoring	1	2	3	4	5	N/A
As a mentee: ability to negotiate and maintain a mentoring relationship	1	2	3	4	5	N/A
As a mentee: ability to receive and incorporate feedback from mentor	1	2	3	4	5	N/A
As a mentor: ability to mentor others	1	2	3	4	5	N/A

Research Skills:

Designing and conducting a research project	1	2	3	4	5	N/A
Program development and evaluation	1	2	3	4	5	N/A
Problem solving/ troubleshooting	1	2	3	4	5	N/A
IRB Submission	1	2	3	4	5	N/A
Analytical skills	1	2	3	4	5	N/A
Computer technology skills (using databases, survey instruments, etc…)	1	2	3	4	5	N/A
Creativity in developing new research directions	1	2	3	4	5	N/A

Scholarship Skills:

Manuscript writing skills	1	2	3	4	5	N/A
Understanding authorship, publication, and integrity	1	2	3	4	5	N/A
Disseminating your work (identifying outlets for publication/presentation)	1	2	3	4	5	N/A
Grant writing skills	1	2	3	4	5	N/A
Finding funding opportunities	1	2	3	4	5	N/A
Responding to reviewers' critiques and revision	1	2	3	4	5	N/A
Creating a research presentation (lecture, poster, etc…)	1	2	3	4	5	N/A

Leadership and Management Skills:

Leading and motivating others (in teams, meetings, committees)	1	2	3	4	5	N/A
Creating and managing a budget	1	2	3	4	5	N/A
Managing projects and programs	1	2	3	4	5	N/A
Time management skills (e.g., workload, planning, pace of career)	1	2	3	4	5	N/A
Organizational skills	1	2	3	4	5	N/A

Interpersonal Skills:

Getting along with others (conflict resolution skills)	1	2	3	4	5	N/A
Giving and receiving feedback	1	2	3	4	5	N/A
Communicating clearly in writing	1	2	3	4	5	N/A
Communicating clearly in conversation	1	2	3	4	5	N/A

Career Development:

Understanding promotion & tenure criteria	1	2	3	4	5	N/A
Clear direction in achieving promotion & tenure requirements	1	2	3	4	5	N/A
CV preparation skills	1	2	3	4	5	N/A
Dossier preparation skills	1	2	3	4	5	N/A
Navigating the organizational/institutional culture	1	2	3	4	5	N/A
Enhancing professional visibility (local and national organizations)	1	2	3	4	5	N/A
Negotiating skills (negotiating for what you want or need)	1	2	3	4	5	N/A
Networking skills (and creating professional networks)	1	2	3	4	5	N/A
Work-life integration skills	1	2	3	4	5	

Teaching Skills:

Small group teaching skills	1	2	3	4	5	N/A
Large group lecture / oral presentation skills	1	2	3	4	5	N/A
Giving feedback to learners	1	2	3	4	5	N/A
Curriculum development	1	2	3	4	5	N/A
