# Supplementary material for: Independent investigator incubator (I3): a comprehensive mentorship program to jumpstart productive research careers for junior faculty
Source: BMC Med Educ. 2018 Aug 6;18:186. doi: 10.1186/s12909-018-1290-3 (PMC6080403; doi:10.1186/s12909-018-1290-3)
Supplement: Supplementary file 2 — Figure S2. Snapshot of mentee performance for I3 mentors. A short report (1 page) was developed to provide the mentors with a “snapshot” of assessment of mentee performance over time in order to supplement mentor impressions of the mentee and to identify perceived deficits that can be addressed. (PDF 6781 kb) [file 12909_2018_1290_MOESM2_ESM.pdf]

SCHOOL OF MEDICINE

IMAGE COMING SOON

John Spence, Ph.D.
Research Associate
Dept. of Pediatrics

What are your current short-term goals?

1. Build local collaborations to expand research program
2. Develop an R21 application and establish an animal surgery paradigm
3. Develop an RO1 application for submission in June 2017

	Mentee Name	Mentee Name
Mentor	Mentor Name	Mentor Name
Start Date (I3 Program)	11.19.15	11.1.16
Date	11.1.16	11.15.17
Months in Program	12 months	24 months
Question: How often do you meet with your mentor?	biweekly	Monthly/as needed
Question: Do you use other I3 resources (grant writer, biostatistician)?	Grant Writer	Grant Writer
Question (1=strongly disagree; 5=strongly agree) 6=N/A		
I had adequate input into the process of being paired	4	4
I am satisfied with the choice of my mentor.	5	5
My mentor was readily available and approachable	5	5
I proactively worked to arrange meetings and manage	5	5
The frequency of our meetings is appropriate for my	5	5
My mentor provides valuable feedback on my work.	5	5
My mentoring relationship is meeting my expectations.	5	5
I would like the mentoring relationship to continue.	5	5
Note: 3=neutral; 4= somewhat satisfied; 5=very satisfied		
satisfaction with your career in academic medicine	4	4
I want my survey responses shared with my mentor	Yes	Yes
Did the I3 program help you achieve speific short-term goals?	Yes	Yes
	2015-2016	2016-2017
co-author	0	3
first author	0	2
corresponding author	1	0
Total Publications	1	5
Funded PI or co-PI		
	R21 (PI) - $422587	R01 (PI) - $2295513
Submitted (PI or co-PI)		
		R21 (co-PI) - $455688
Ongoing Funding		
	AHA-CDA (PI) - $308000	R21 (PI) - $422587
		AHA-CDA (PI) - $308000

**Research Skills:**	
Designing and conducting a research project	2
Program development and evaluation	6
Problem solving/ troubleshooting	3
IRB Submission	3
Analytical skills	4
Computer technology skills	2
Creativity in developing new research directions	2
**Scholarship Skills:**	
Manuscript writing skills	5
Understanding authorship, publication, and integrity	5
Disseminating your work	5
Grant writing skills	2
Finding funding opportunities	2
Responding to reviewers' critiques and revision	5
Creating a research presentation	5
**Leadership and Management Skills:**	
Leading and motivating others	3
Creating and managing a budget	1
Managing projects and programs	1
Time management skills	2
Organizational skills	5
**Interpersonal Skills:**	
Getting along with others	5
Giving and receiving feedback	4
Communicating clearly in writing	5
Communicating clearly in conversation	
**Career Development:**	
Understanding promotion & tenure criteria	2
Clear direction in achieving promotion & tenure	2
CV preparation skills	2
Dossier preparation skills	2
Navigating the organizational/institutional culture	1
Enhancing professional visibility	4
Negotiating skills	1
Networking skills	2
Work-life integration skills	2
